# Infection with Influenzavirus A in a murine model induces epithelial bronchial lesions and distinct waves of innate immune-cell recruitment

**DOI:** 10.3389/fimmu.2023.1241323

**Published:** 2023-08-15

**Authors:** Frédéric Rivière, Julien Burger, François Lefèvre, Annabelle Garnier, Clarisse Vigne, Jean-Nicolas Tournier, Emmanuelle Billon-Denis

**Affiliations:** ^1^ Immunity and Pathogen Unit, Microbiology and Infectious Diseases Department, Institut de Recherche Biomédicale des Armées (IRBA), Brétigny-sur-Orge, France; ^2^ Respiratory Department, Percy Military Teaching Hospital, Clamart, France; ^3^ Ecole du Val-de-Grâce, Paris, France; ^4^ Institut National de Recherche pour l’Agriculture, l’Alimentation et l’Environnement (INRAE), Unité de Recherche (UR) 0892 Virology and Molecular Immunology Unit, Centre de recherche Ile-de-France-Jouy-en-Josas, Jouy-en-Josas, France; ^5^ Innovative Vaccine Laboratory, Institut Pasteur, Paris, France

**Keywords:** two-photon excitation microscopy, Influenza virus, immune response, flow cytometry, cytokines, live lung slice

## Abstract

**Introduction:**

Inflammatory lesions after Influenza A viruses (IAV) are potential therapeutic target for which better understanding of post-infection immune mechanisms is required. Most studies to evaluate innate immune reactions induced by IAV are based on quantitative/functional methods and anatomical exploration is most often non-existent. We aimed to study pulmonary damage and macrophage recruitment using two-photon excitation microscopy (TPEM) after IAV infection.

**Methods:**

We infected C57BL/6 CD11c^+^YFP mice with A/Puerto Ricco/8/34 H1N1. We performed immune cell analysis, including flow cytometry, cytokine concentration assays, and TPEM observations after staining with anti-F4/80 antibody coupled to BV421. We adapted live lung slice (LLS) method for *ex-vivo* intravital microscopy to analyze cell motility.

**Results:**

TPEM provided complementary data to flow cytometry and cytokine assays by allowing observation of bronchial epithelium lesions and spreading of local infection. Addition of F4/80-BV421 staining allowed us to precisely determine timing of recruitment and pulmonary migration of macrophages. *Ex-vivo* LLS preserved cellular viability, allowing us to observe acceleration of macrophage motility.

**Conclusion:**

After IAV infection, we were able to explore structural consequences and successive waves of innate immune cell recruitment. By combining microscopy, flow cytometry and chemokine measurements, we describe novel and precise scenario of innate immune response against IAV.

## 1 Introduction

Influenza A viruses (IAVs) cause potentially life-threatening respiratory disease (1–3). It is sometimes difficult to predict the severity of the outcome following infection, which presents a challenge for both disease prevention and the treatment of infected patients. The symptoms of IAV infection (‘flu’) range from asymptomatic to fatal, and it has an estimated worldwide burden of between 300,000 and 650,000 deaths annually (1). The efficacy of oseltamivir for severe forms is still debated (4).

A recent meta-analysis indicated that non-steroidal anti-inflammatory drugs (NSAIDs) are effective in reducing mortality following influenza infection (5). The treatment of inflammation *per se* and targeting the mechanisms of innate immunity following an infection may offer new therapeutic targets. Data from animal studies have shown that post IAV-infection cytokine storms, followed by an influx of macrophages and neutrophils, negatively affect survival if alveolar macrophages are depleted (6, 7).

The methods used for most studies, to date, have consisted of conventional cellular, biochemical, and histological analysis techniques (8–10). Two-photon excitation microscopy (TPEM) is now widespread in biological safety laboratories, allowing the dynamic study of cell-pathogen interactions with good resolution, improving study outcomes (11). TPEM has been difficult to apply to the lung due to physiological movement within the thorax (12, 13). However, TPEM has been used with a limited number of pathogens to analyse pulmonary infections with *Bacillus anthracis* and pulmonary viruses, such as SARS-CoV-2 and IAVs (14–17). Most studies using TPEM on IAV have focused on the role of neutrophils, as they are the first cell line in the defense of the lungs (18). It is now possible to obtain both two- and three-dimensional images using *ex vivo* and *in vivo* protocols, with time-lapse analysis allowing a fourth dimension. Another advantage of TPEM is that two-photon excitation labels non-centrosymmetric tissues, such as collagen, by the generation of a second harmonic signal, without the need for additional staining (14, 19–21). Intravital imaging can be performed with TPEM using various protocols, including *in vivo*, explanted lung, *ex viv*o organ analysis to observe cell motility (22).

We aimed to explore the anatomical damage and immunological response after IAV infection using TPEM. Here, we describe early anatomical pulmonary lesions and the precise dynamics of cellular recruitment of monocyte-derived macrophages using TPEM by *ex-vivo* intravital imaging. By combining microscopy, flow cytometry, and cytokine/chemokine measurements, we describe a precise scenario of the innate immune response against IAV.

## 2 Materials and methods

### 2.1 Ethical approval

All animal care and experiments conformed to the requirements of the Animal Research Committee of the French Military Health Service (authorized project with number 2020-11), as well as the European Parliament directive 2010/63/EU guidelines for the protection of animals used for scientific purposes.

### 2.2 Animal models

Wildtype C57BL/6 mice were used to study the pathophysiology of IAV infection (Rockefeller University, New York, USA). In total, 46 male mice were used, divided into three groups of 12 animals, one group each for flow cytometry, cytokine concentration measurements, and TPEM on 4% paraformaldehyde (PFA) fixed lungs, and 10 animals for *ex vivo* intravital imaging by TPEM. All animals were handled and housed under specific pathogen-free conditions. Their bodyweight was recorded daily. All ethical issues related to mouse experimentation were carefully considered.

The preliminary studies of F. Lefevre’s laboratory on fluorescent virus were carried out only on male mice. In order to have a homogeneous and comparable population, to fulfil ethical guidelines of European directive in terms of animal reduction, we deliberately included only male mice. Otherwise, to have a sufficient and statistically comparable population between gender, it would have required many more animals.

Mice used for flow cytometry and the cytokine concentration measurements were 6 ± 2 weeks of age. For the TPEM group, the 22 transgenic CD11c+^YFP^ mice (on a C57BL/6 genetic background, bred to express yellow fluorescent protein (YFP) in lung macrophages under control of the CD11c promoter) were six to eight weeks of age (23).

### 2.3 Influenza A virus strains and reverse genetic construction

We used the IAV A/Puerto Rico/8/34/H1N1 (PR8) strain for infections followed by cytometry and cytokine measurements. For the TPEM experiments, the fluorescent viral strain A/Puerto Rico/8/34/H1N1 subtype expressing red fluorescent protein (RFP) fused to the NS1 protein (RFP-NS1-PR8) was created using reverse genetics (F. Lefèvre, non-published data). Briefly, the RFP-NS1-PR8 strain was constructed with RFP using the same protocol as previously described for GFP by Garcia-Sastre et al. (24) (25–27). All experiments were performed in a biosafety level 2 laboratory.

### 2.4 Infection with the influenza virus

Prior to experimental infection on day (D)0, all mice were anaesthetized with 5% isoflurane. They were then intranasally instilled with 30 µL phosphate buffered saline (PBS) containing either 10^5^ plaque-forming units of RFP-NS1-PR8 (TPEM group) or PR8 (flow cytometry and cytokine groups). Three animals from each group of 12 mice were not infected as controls: they underwent the same procedures but received only a PBS solution. The control and experimental groups of animals were housed in separated isolators and monitored and their bodyweight recorded daily on D1, D2, D3, and D4. All experiments were performed in a biosafety level 2 animal facility.

### 2.5 Tissue preparation

On D1, D2, D3, and D4 post-infection, one mouse from each of the three groups (flow cytometry, cytokine, and TPEM) were euthanized by cervical dislocation. Tissue samples were prepared following intracardiac perfusion of 10 ml PBS containing unfractionated heparin (10 International Unit (IU)/mL). For the flow cytometry and cytokine samples, this was followed by perfusion with10 mL PBS. For the TPEM group, 10 mL 4% paraformaldehyde (PFA) solution was perfused instead.

### 2.6 Preparation of slices for *ex vivo* imaging by TPEM (lung live slice without agarose)

The protocol is described in **Supplementary Data**.

### 2.7 TPEM imaging

In the TPEM group, the left lobe from each set of harvested lungs was glued onto a Vibratome^®^ stage (Leica, Wetzlar, Germany) and immersed in PBS. To obtain a smooth lung section, the lobe was cut into two 10 μm/s-thick slices using a razor blade with a vibration amplitude of 3 mm. The lower section (4 mm thick) was glued onto an 83 mm petri dish and immersed in PBS before imaging.

TPEM was performed using an LSM 710 NLO microscope (Carl Zeiss) equipped with an infrared laser coupled to an optical parametric oscillator (Chameleon; Coherent). The wavelength of the first laser was set to 850 nm for the excitation of YFP, second harmonic, and blue BV421 (‘Brilliant Violet’, Bio Legend^®^) markers. The wavelength of the second laser was set to 1,100 nm for the viral RFP. Each control and infected lung section was fully explored in both the x and y-axes for a thickness of 100 µm in the z-axis to map the most representative portion of the sample. The microscope was set up in a biosafety level 2 laboratory.

To stain macrophages, lung sections were incubated in PBS with Fc Block (1/300^e^) for 30 min at room temperature for PFA fixed lungs and at 37°C for the other protocols for *ex vivo* imaging. Lung sections were incubated in PBS containing F4/80 antibodies (specifically recognizing the macrophage population) coupled to the BV421 fluorescent marker (1/100^e^) for 2 h at room temperature for PFA fixed lungs and at 37°C for the other protocols for *ex vivo* imaging.

### 2.8 Measurement of cell speed and distance travelled

The slices were maintained at 37°C in a temperature-controlled chamber. The analysis was based on cell morphology as previously published for several types of fluorescent reporters (28, 29). We manually separated the CD11c^+^ cells with multiple dendritic projections (named dendritic-shaped cells [DSCs] [dendritic cells]) and CD11c^+^ or CD11c^low^ round cells (named round-shaped cells [RSCs] [resident macrophages and monocyte derivatives]) under microscopy (28). The distance and speed of displacement were measured by manually focusing on the center of each cell in each plane for all videos made at the same time post-infection (according to the acquisition time to obtain the speed).

### 2.9 Flow cytometry analysis

The mouse lung monocyte and macrophage populations were divided into several subpopulations, including alveolar macrophages (CD11c^high^, F4/80^+^), interstitial macrophages (CD11c^low^, F4/80^+^), and resident monocytes (CD11c^low^, F4/80^+^) as previously described (6, 26). As our aim was to explore all macrophage populations, including resident, interstitial, and migratory macrophages, we selected the F4/80 marker, which is common to all macrophage subpopulations.

The flow cytometry protocol is described in **Supplementary Data**.

### 2.10 Cytokine concentration quantification

Cytokine concentrations were measured using multiplexed sandwich ELISA-based quantitative array platforms of whole lung samples. For each mouse in this group, both lungs were removed and homogenized using a gentleMACS™ dissociator (as above). Each whole lung lysate was prepared according to the proprietary array kit protocol (Tebu-Bio, Quantibody^®^ Mouse Inflammation Array Kit, Manufacturer REF, 40 cytokines/chemokines). Each sample was plated in duplicate and incubated with a standard cytokine mix, an antibody detection cocktail, and, finally, Cy3-equivalent dye-conjugated streptavidin before the plate was dried and read.

### 2.11 Statistical analysis

The results for quantitative variables are expressed as the mean and standard deviation. Data were collected and organized using Excel (Microsoft Corp., Washington, USA). Statistical analyses were performed using R software (The R Foundation, version 3.1.3). Comparisons between control and experimental samples were performed using Student’s T test for quantitative variables; the results were considered statistically significant for p<0.05.

## 3 Results

### 3.1 Post-infection pulmonary cell recruitment

**Figure 1:**
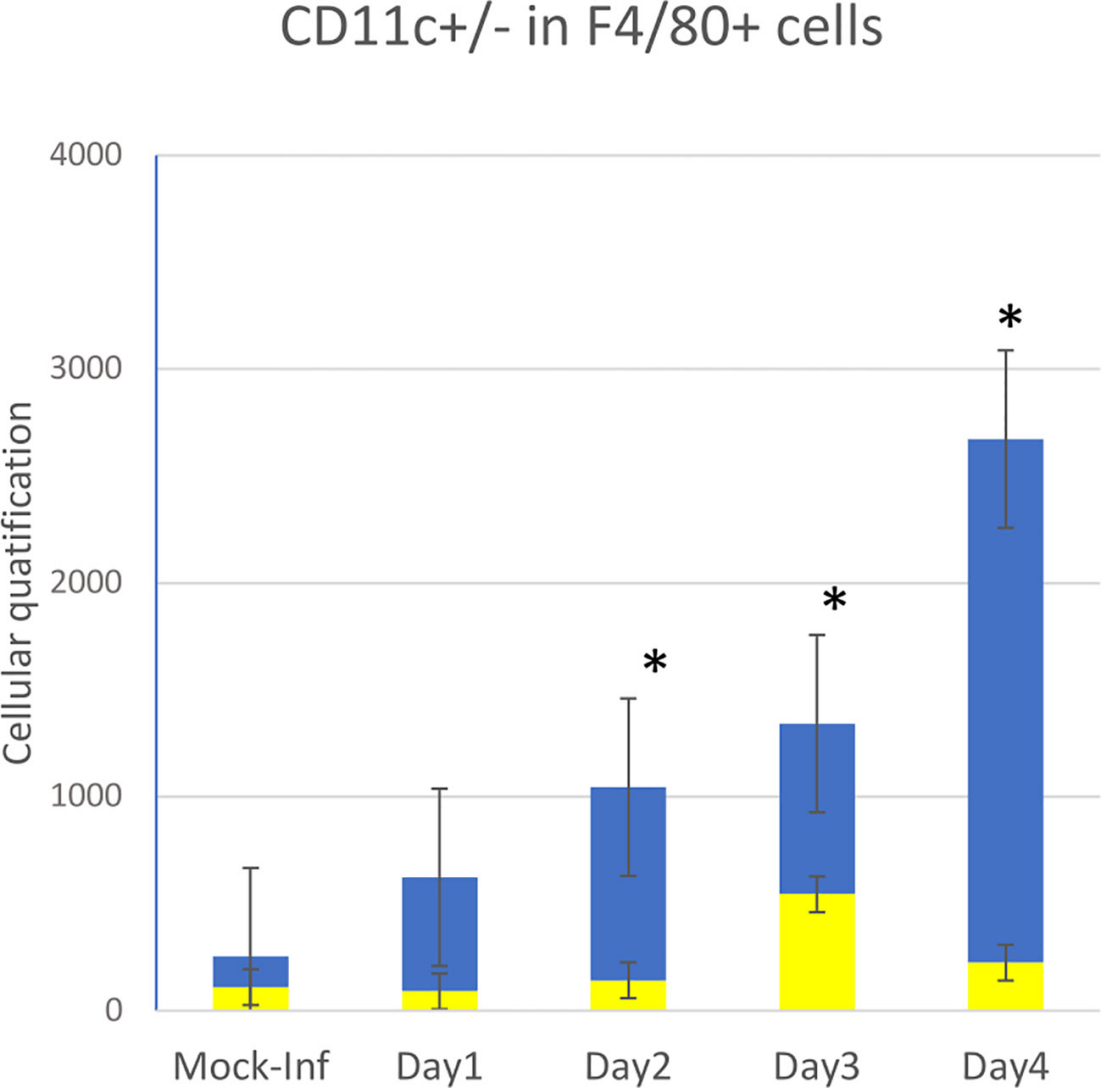
Flow cytometry data showing quantitative data and the evolution of innate immune cell recruitment in whole-lung tissues of 10 mice infected on D0 with influenza A (PR8 strain) versus that of two non-infected control animal(s) (mock-inf = mock-infected). Four types of cells were measured by flow cytometry: repartition in F4/80+ cells between CD11c+ (yellow bar) and CDIl c- (blue bar), *p<0.05.

We focused on major myeloid immune cell types using the macrophage markers F4/80 and CD11c (**Figure 1**).

As previously described, the first cells recruited to the lung following infection with IAV PR8 on D0 were granulocytes (Ly6G^+^). The number of granulocytes rapidly and significantly increased in the infected group during the first day post-infection (p>0.05), peaking on D2, and then rapidly decreased on D3 and D4 back to basal levels (data not shown).

CD11c^+^ cells were then recruited, consisting mostly of monocytes, which peaked on D3. The number of pulmonary macrophages (F4/80^+^) increased slowly between days 1 and 2 (p<0.05), reaching a peak at D4 (four times higher by D4). Over the same period, myeloid cells remained at basal levels in the control animals.

Within the F4/80^+^ population, the number of CD11c^+^ cells remained stable over time post-infection, without a significant difference from that of mock-infected mice. The number of CD11c^-^ cells progressively and significantly increased from D1 to D4 relative to that of mock-infected animals, more significantly between D3 and D4.

### 3.2 Whole-lung cytokine concentrations post-infection

**Figure 2:**
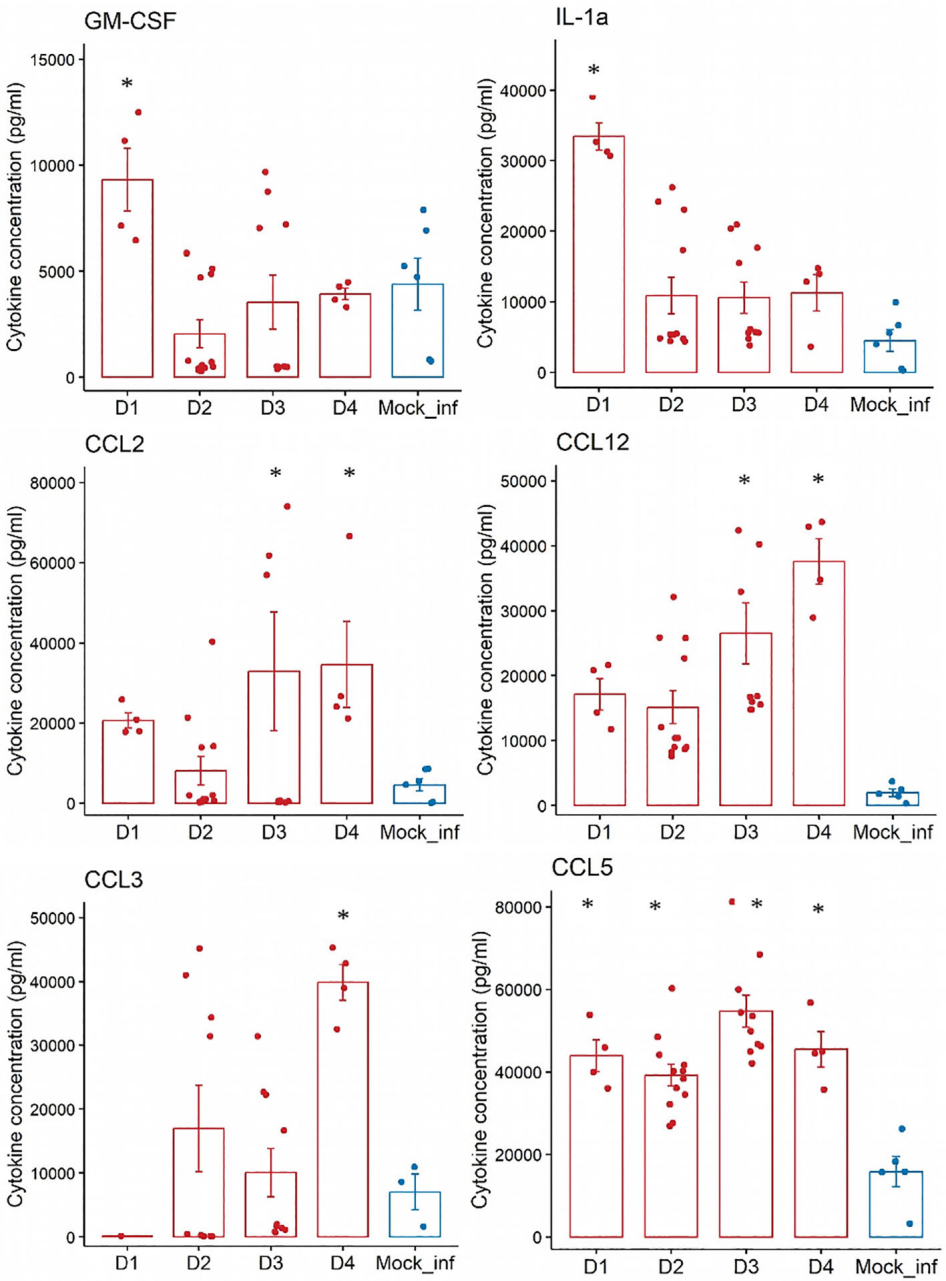
Analyses of the concentrations (pg/mL) of nine proinflammatory cytokines and chemokines in whole-lung samples from n=10 mice experimentally infected at day 0 with influenza A (PR8) versus a/n=2 control animal(s) that did not receive any infection over a 4-days experiment. Cytokines: GM-CSF, IL-1a. Chemokines: CCL2, CCL12, CCL3 and CCL5. mock-infected = blue bar; Infected with PR8= red bar- *:p<0.05 (vs mock-infected).

We chose to report here only the results of the more relevant proinflammatory cytokines and chemokines (GM-CSF, IL-1a, CCL2, CCL12, CCL3, and CCL5) in macrophage recruitment secreted in whole lung samples from both control and infected mice (**Figure 2**). GM-CSF and IL-1a concentrations peaked on D1 (p<0.05 *vs*. control) before decreasing on D2.

The concentration of CCL5 peaked on D1 (p<0.05 *vs* mock-infected) and those of CCL2 and CCL12 peaked on D3 (p<0.05 *vs* mock-infected). The concentration of CCL3 peaked later, on D4 (p<0.05 *vs* control).

The dynamics of CCL2 and CCL12 secretion likely reflect the recruitment of monocytes and those of CCL3 the later wave of CD11c^+^ dendritic cells and macrophages. The cytokine IL-7, which also plays a role in the recruitment and migration of innate immune cells, reached its peak by D2 post-infection (p<0.05 *vs* control), before its concentration plateaued.

### 3.3 TPEM observations on fixed lungs

**Figure 3:**
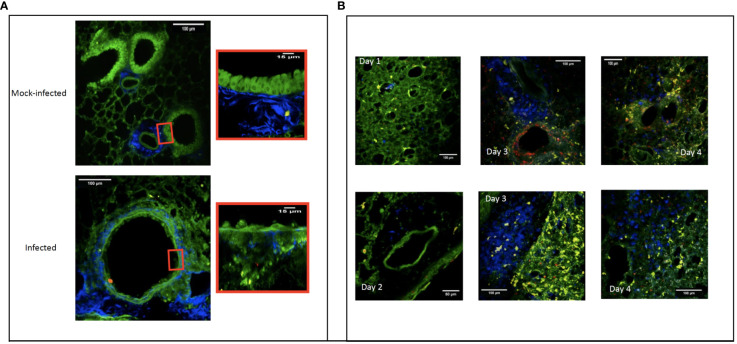
Two-photon excitation micrographs showing the pulmonary bronchial epithelium three days after experimental infection with a modified influenza A/Puerto Rico/8/34/H1N1 strain that expresses red fluorescent protein. The replicating virus is shown in red and the epithelial cells in green. Image showing the normal bronchiolar epithelium of mock-infected mice and a zoom of normal epithelium versus a zoom of infected bronchiolar epithelium **(A)**. Connective tissue (fiber in blue), macrophages (round cells in blue), CD11C^+^ cells (yellow). Recruitment to the mouse lung over four days following infection with a modified influenza A/Puerto Rico/8/34/H1N1 strain expressing red fluorescent protein. On D1 post-infection, resident macrophages can be seen (shown in blue) in the lung parenchyma. On D2, the macrophages have not yet moved into the perivascular areas. By D3, they can be seen in the perivascular area near the infected (red) bronchial epithelium. Finally, on D4, the macrophages have become homogeneously and diffusely distributed throughout the whole lung section. CD11c^+^ cells (yellow), bronchial and alveolar epithelium (green) **(B)**.

We next examined the correlation between cell recruitment after infection and the anatomical lesions observed by TPEM. We used the second harmonic generation signal to image the collagen in bronchial structures and the green autofluorescence of bronchial cells to image bronchial cells without staining. The TPEM micrographs of the control group showed them to have healthy lungs, with intact bronchial and alveolar epithelia (shown in green) and connective tissue (in blue) (**Figure 3A**, mock-infected).

Over three days, the virus (shown in red) replicated first in the bronchial epithelium, followed by replication in the alveolar epithelium (**Figure 3** – panel A). The observed epithelial infection led to destruction of the bronchial and bronchiolar epithelia. By D4, macrophages (shown as blue cells) were also visible in the basal membrane (**Figure 3A**).

The addition of an anti-F4/80 antibody coupled to the fluorochrome BV421 allowed us to follow the recruitment of F4/80^+^ macrophages (shown in blue) over time. In the early phase of the infection (days 1 and 2), more F4/80^+^ macrophages were visible in infected lungs than mock-infected lungs. Macrophages were homogeneously distributed within the parenchyma, but absent from the perivascular zones. On D3, we observed a high density of F4/80^+^ macrophages in the perivascular regions. By D4, F4/80^+^ macrophages were densely and homogeneously disseminated throughout the tissue of the entire lung (**Figure 3B**).

We also observed the scavenger role of F4/80^+^ macrophages, as they phagocytosed virus-infected cells or cell fragments. This suggests that F4/80^+^ macrophages were first recruited to the lung in the perivascular spaces and peribronchiolar area and then to the lung parenchyma to participate in lung homeostasis by scavenging infected cells and cell fragments.

### 3.4 *Ex vivo* cell dynamics by live lung slice imaging TPEM

Finally, we used TPEM to address the cellular dynamics after IAV infection. The distance traveled by the dendritic-like DSCs of the infected lungs was significantly greater at all time points post-infection (D1, D2, D3, and D4) than that of DSCs of the control lungs (1.54 ± 1.03 *μ*m) (p<10^-4^ for all time points post-infection) (**Table 1**). The distance traveled was stable, with no significant difference depending on the day post-infection (p>0.05).

**Table 1:** Average distance travelled by DSCs and RSCs according to time post infection.

Time (day)		Distance of DSCs in *μ*Am, mean ± SD (number of cells)	Distance of RSCs in *μ*m, mean ± SD (number of cells)	p
	Day 1 (N)	5,33 ± 2,65 (22)	26,47 ± 10,54 (14)	<10^-5^
	Day 2 (N)	5,03 ± 3,69 (18)	9,76 ± 4,57 (15)	0,002
	Day 3 (N)	7,69 ± 4,14 (17)	23,37 ± 11,42 (16)	0,001
	Day 4 (N)	5,69 ± 5,59 (22)	22,04 ± 11,94 (26)	<10^-5^
	Mock-infected (N)	1,54 ± 1,03 (5)	2,8126 ± 1,76 (5)	0,105

Time (day)		Speed of DSCs in *μ*m/min, mean ± SD	Distance of RSCs in *μ*m, mean ± SD (number of cells)	p
	Mock-infected (N)	0,10 ± 0,0488	0,09 ± 0,081	0,138
	Day 1 (N)	0,14 ± 0,098	0,62 ± 0,227	<10-4
	Day 2 (N)	0,11 ± 0,055	0,3 ± 0,145	0,0012
	Day 3 (N)	0,12 ± 0,085	0,37 ± 0,156	<10-4
	Day 4 (N)	0,045 ± 0,031	0,62 ± 0,211	<10-5

Significance if p<0.05 (student test). Movement speed of dendritic-like cells and round-shaped cells as a function of time after PR8 infection. Significance if p<0.05 (student test). D, day; Post inf, post-infection; Mock-Inf, uninfected mice; DSC, Dendritic-shaped cell, RSC, Rounded-shaped cell; N, number of cells that can be used for the calculation.

The distance traveled by RSCs of infected lungs was also significantly greater than that of RSCs of mock-infected lung cells at all time points post-infection (D1, D2, D3, and D4) (2.8126 ± 1.76 *μ*m) (p<10^-4^ for all time points post-infection). Interestingly, on D2, the distance traveled was significantly lower (9.76 ± 4.57 *μ*m) than on the other days post-infection. The distance travelled was stable between D1, D3, and D4, with no significant difference (p>0.05).

Overall, the distances traveled by DSCs and RSCs for a given time point post-infection were no different in the control lungs, whereas the distance travelled by RSCs was significantly greater than that of DSCs for each time point post-infection in infected lungs.

The speed of the movement of the DSCs of the infected lungs was higher at all times post-infection (D1, D2, D3, and D4) than that of DSCs of the control lungs (p<0.003 for all time points post-infection). However, we observed no difference in their speed between time points post-infection (p>0.05).

Similar to the DSCs, the speed of the movement of RSCs was significantly higher at all times post-infection (D1, D2, D3 and D4) than that of RSCs of the control mock-infected lungs (2.8126 ± 1.76 *μ*m/min) (p<0.004 for all times). We observed early acceleration of their movement from D1 (0.09 ± 0.081 *μ*m/min) (*vs* control, p<10^-5^) and then the same speed for D2, D3, and D4 (without a significant difference between D1, D2, D3, and D4).

Overall, the speed of movement of the DSCs and RSCs for a given time point post-infection was no different in the control lungs, whereas the speed of movement of RSCs was significantly higher than that of DSCs for each time point post-infection.

**Figure 4:**
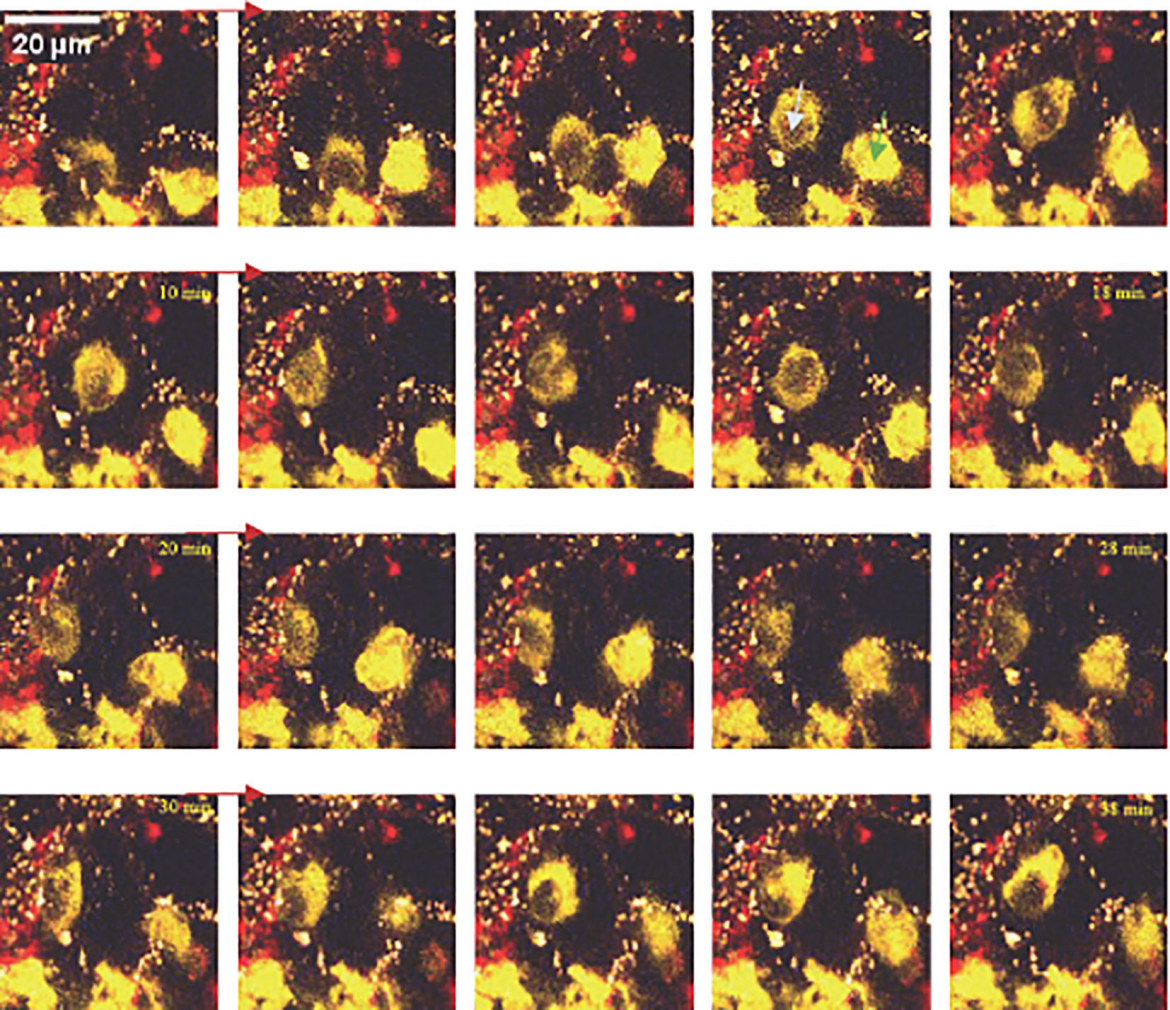
Continuous successive images acquired by TPEM *ex vivo* of a mouse lung at D1 post-infection. Inter-alveolar motility of two round cells (macrophages), one macroscopically CD11c^+^ (image 4), the other CD11c^low^ (image 4), interacting with each other via direct contact.

The phenotypic distinction between RSCs and DSCs is illustrated in the videos in **Figure 4**, as well as the preservation of cell motility in our modified LLS protocol. This allowed visualization of the interactions of mobile round cells (probably macrophages) and infected cells, the cell migration of probable macrophages after contact with infected cells, and the likely direct interaction between macrophages. Videos 1 and 2 (**Supplementary Datas 1, 2**) show poorly mobile CD11c^+^ cells in the context of immunological monitoring, allowing them to quickly interact with infected epithelial cells, multiplying the cellular interactions during infection.

## 4 Discussion

### 4.1 Epithelial injuries

The study of the evolution of infected sites by TPEM showed that IAV initially infected bronchial and bronchiolar epithelial cells (D1) in our model, before spreading to the alveolar epithelium on D3. This study provides novel data describing the evolution of IAV respiratory infection and its progression, leading to acute respiratory failure.

The observation of epithelial lesions is of paramount interest because they reflect the cytopathogenic effect of the virus on infected structures and provide an anatomical explanation for the symptomatology of bronchitis following IAV infection (3). The development of such lesions is clearly among the mechanisms to explain the frequency of bacterial superinfections due to the loss of the mechanical barrier provided by the epithelium and mucociliary layer. Our study is among the first to describe IAV-induced epithelial lesions by TPEM except for partial data from artificial cell culture *ex vivo* models (30). We also observed the presence of F4/80^+^ cells on D4, which were most likely monocyte-derived macrophages (MDMs), forming a “cell carpet” over the basal membrane of the damaged epithelium. This could be explained by the prominent role of scavenger or “garbage collector” MDMs phagocytosing the apoptotic and necrotic epithelial cells. We cannot exclude that this “cell carpet” may also serve a protective function for the unprotected basement membrane by creating a substitute mechanical barrier before regeneration of the epithelium. These two hypotheses can be considered as the beginning of the cell repair process and/or a protective mechanism against bacterial superinfections. The questions arising from this observation merit a more specific study.

### 4.2 Macrophages recruitment

As described in the literature, we observed marked granulocyte recruitment, followed by that of F4/80^+^ macrophages (31). The resident macrophages (F4/80^+^ and CD11c^+^) remained quantitatively stable during the post-infection period, whereas the recruited cells were composed of F4/80^+^ CD11c^-^ cells, likely corresponding to the differentiation of circulating monocytes into MDMs migrating to the lung at the infected site. It is, therefore, likely that the influx of F4/80^+^ cells observable by TPEM corresponds to that of MDMs. However, their recruitment appears to be too rapid and too early to be the consequence of chemotaxis via CCL2, as the assays only showed an increase in its concentration in the lung from D2. It is possible that these MDMs were derived from undifferentiated (F4/80^-^) “sentinel” monocytes circulating in the lungs in the normal state, as described by Rodero et al. (32), which then differentiated into MDMs (F4/80^+^, CD11c^-^) under the action of GM-CSF present on D1. Subsequently, monocytes following a chemokine gradient (CCL2 and CCL12, CXCL9, and CCL3) likely migrated to the lungs and differentiated into MDMs. This phenomenon is illustrated in our study by TPEM from D3 post-infection, with the observation of the perivascular infiltration of F4/80^+^ cells. This indicates the intra-pulmonary migration of these monocytes via blood circulation, which then differentiate into MDMs (F4/80^+^ CD11c^-^ cytometry data). These MDMs then migrate throughout the lung parenchyma.

### 4.3 *Ex vivo* intravital imaging

For intravital imaging, *in vivo* methods are the most common and physiologically relevant tools, allowing the analysis of all anatomical components, in particular, the vasculature (17). However, this requires anesthesia, surgical skill with small animals, and an adequate technical platform. In addition, they only explore the subpleural (100 µm deep) area under the thoracic window (22, 33). *Ex-vivo* techniques have been developed, in particular, the LLS method (12). This technique is most often used in culture after the fragmentation of slices from lung lobes (34–36) and secondary incubation with the infecting agent. To be as physiological as possible, we eliminated the step of liquefied agarose intra-tracheal instillation. In our hands, this did not affect the ease of performing the vibratome cuts. It allowed us to study IAV infection under physiological conditions until the moment of euthanizing the animals and preparing the organs for imaging. Our results thus confirm the feasibility and relevance of this agarose-free technique for the *ex-vivo* imaging of IAV infection in the lungs.

Although we found no difference in either the speed or distance travelled between DSCs and RSCs in the basal state (control), these two cell types moved significantly more and faster after infection. After infection, the significantly greater distance and speed of movement of RSCs than DSCs appears to validate the separation of these cell types based on their morphological differences. This provided functional motility data on cell recruitment in the context of infection. The motility of these cells induced by viral infection generated interactions between the macrophage-type cells (macrophages, DCs, MDMs) and infected cells, observable by TPEM.

### 4.4 Synthesis

**Figure 5:**
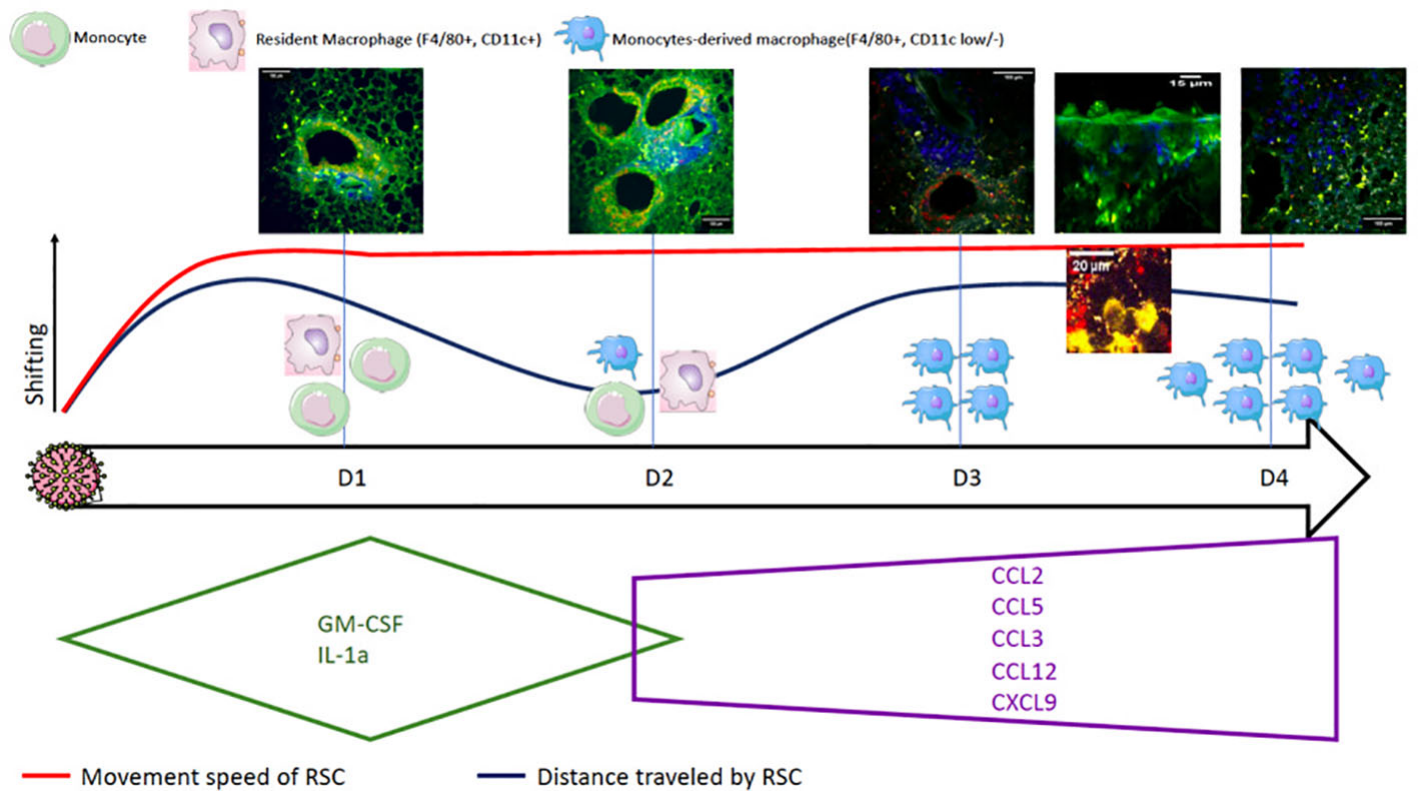
Summary diagram of the pulmonary consequences after infection with *Influenzavirus* in a mouse model by compiling data from flow cytometry, cytokine assays, and TPEM on fixed and *ex vivo* lungs.

Compilation of the data from cytometry, cytokine assays, and TPEM on fixed lungs and *ex-vivo* imaging allows us to propose the following scenario (**Figure 5**). (i) In the basal state, resident macrophages and DCs are on immunological standby (32, 37). (ii) After infection, the infected epithelial cells secrete GM-CSF in the early phase (D1 to D2) to rapidly activate resident macrophages and initiate the differentiation of sentinel monocytes (38) into macrophages (MDMs). (iii) Such activation leads to lifting of the immunological standby phase, resulting in an increase in motility of the resident macrophages, MDMs, and, to a lesser extent, DCs. It also provokes the acceleration of their movement, allowing them to multiply cellular interactions (phagocytosis, antigen presentation, etc.). (iv) The D2 time point can possibly be considered as a transition from the production of pro-inflammatory cytokines, which begins to decrease, to that of chemokines, which participate in the second wave of macrophage recruitment; this could explain the shorter distance traveled by the first recruited macrophages positioned at the infected site. (v) A second phase appears on D3 by chemotaxis via CCL-2 (35, 38), in which circulating monocytes enter through the vascular route, differentiate into MDMs, and then migrate and diffuse throughout the lungs. This activation is maintained by the macrophages themselves, which secrete the pro-inflammatory cytokines necessary for their stimulation, inducing increased motility. The migration of the recruited MDMs to the infected site could likely explain the second increase in the distance traveled by RSCs while maintaining a high speed of movement.

The evolution of cytokine secretion allowed for a better understanding of the massive cell recruitment observed by TPEM. The cytokines produced in the very early phase (D1) (GM-CSF and IL-1a) ensure the activation of DCs and macrophages on immunological standby. In a second step, on D3, the molecules CCL2, CCL12, CXCL9, CCL3, and CCL5, produced, in particular, by the activated macrophages, maintain cell recruitment and differentiation, in particular, that of MDMs. Finally, in the late phase on D4, the production of cytokines that activate and regulate innate immunity begins, inducing the inhibition of macrophages and DCs.

In conclusion, TPEM allowed for a better understanding of the viral physiopathology of IAV infection, showing the appearance of epithelial lesions and highlighting different stages of macrophage recruitment (early (D1)-transition (D2)-late (D3-4)). This should encourage continued research on the recruitment of innate immune cells, which could be a therapeutic target, in particular, of anti-inflammatory drugs and other immunomodulator treatment.

## Data availability statement

The original contributions presented in the study are included in the article/**Supplementary Material**. Further inquiries can be directed to the corresponding author.

## Ethics statement

The animal study was approved by Animal Research Committee of the French Military Health Service (authorized project with number 2020-11). The study was conducted in accordance with the local legislation and institutional requirements.

## Author contributions

Conception: EB-D and FR. Data collection: FR, JB and AG. Analysis: FR and EB-D. Writing: FR. Critical review and approval: EB-D, JB, FL, AG, and J-NT. All authors contributed to the article and approved the submitted version.
