# Supplementary material for: Infection with Influenzavirus A in a murine model induces epithelial bronchial lesions and distinct waves of innate immune-cell recruitment
Source: Front Immunol. 2023 Aug 15;14:1241323. doi: 10.3389/fimmu.2023.1241323 (PMC10464834; doi:10.3389/fimmu.2023.1241323)
Supplement: Supplementary file 1 [file DataSheet_1.docx]

Flow cytometry protocol

For the cytometry experiments, both cell lungs were harvested and homogenised using a gentleMACS™ dissociator before being incubated with type IV collagenase at 37°C for 10 minutes. Remaining red blood cells were lysed using 1 X red blood cell lysis buffer BD (Bioscience®). The cells were stained with blue Fixable Viability Dye eFluor™ 455 (eBioscience™). Anti-mouse immunophenotyping antibodies were diluted in FACS buffer to 5µg/mL along with Fc Block before the cells were stained for 30 minutes on ice using the following cytokine/antibody/marker solutions: CD45 (BD Biosciences®), CD11b (BD Biosciences®), CD11C (BD Biosciences®), F4/80 (Bio Legend®), CD3 (BD Biosciences®), CD4 (Bio Legend®), CD8 (BD Biosciences®), Ly6G (Bio Legend®), CD103 (BD Biosciences®). After staining, the cells were washed twice with buffer and then fixed in 4% paraformaldehyde in FACS buffer for 15 minutes. Cell numbers were collected using an LSR II flow cytometer (BD Bioscience®, Franklin Lakes, NJ) and the data were interpreted using FlowJo software (Treestar, Ashland, OR). All interpretations were performed after doublet and dead cells exclusion.

F4/80+, CD11c-

F4/80+, CD11c+

F4/80-, CD11c+

Plots to explicate gating protocol to quantify cells CD11c + or – into F4/80+ cells.
