# Supplementary material for: Infection with Influenzavirus A in a murine model induces epithelial bronchial lesions and distinct waves of innate immune-cell recruitment
Source: Front Immunol. 2023 Aug 15;14:1241323. doi: 10.3389/fimmu.2023.1241323 (PMC10464834; doi:10.3389/fimmu.2023.1241323)
Supplement: Supplementary file 2 [file DataSheet_2.docx]

“Modified” Live Lung Slice without agarose protocol

The technique used described below is slightly different from the technique usually published (justification is bellow in the discussion section)(20).

1. Euthanasia of mice by cervical dislocation.

2. Opening of the abdominal cavity, incision and careful dissection of the diaphragm

3. Dissection of the right and left lateral chest walls and exclusion of the thoracic flap.

4. Incision of the right atrium and intracardiac instillation of 2 X 10mL of DMEM without phenol red at 37°C.

5. Dissection of the trachea, inferior vena cava and release of the lung which will be immersed in DMEM without phenol red and transported at 37°C.

6. The left lobe being the most voluminous, it is used for the TPEM experiment. It is glued to the plate of the vibratome (Leica, Nussloch, Germany) and the lobe is sliced ​​into 2 parts at 10 µm/s of vibration amplitude of a razor blade (slice thickness: 3 to 5 mm).

Labelling of the lung section:

1. The lung section is incubated with a Rat Anti-Mouse CD16/CD32 antibody (Mouse BD Fc Block™ - BD biosciences°) diluted at 1/300 for 30 minutes.

2. Three washes with DMEM without phenol red at 37° C. for 5 minutes each with gentle agitation are done.

3. An incubation with Rat Anti-Mouse F4/80-Like Receptor antibody coupled BV421 (BD Biosciences°) diluted at 1/50 for 1 hour is done.

4. Three washes with DMEM without phenol red at 37° C. for 5 minutes each with gentle agitation are done.

5. The section is then glued to a glued slide under the imaging infusion set (Figure 1). The slides and the receptacle are kept in an incubator at 37°C until use.


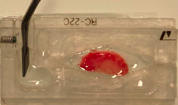


Figure 1 : Lung slice conditioning device for imaging. Slice of lung glued to the center allowing continuous irrigation (hydration-oxygenation) via infusion of DMEM without phenol red at 37°C.

Preparation for TPEM

As shown in the figure 2, the lung section glued to the stage is continuously irrigated by an infusion of DMEM without phenol red at 37°C by a peristaltic pump perfusion-aspiration system. The DMEM is stored in aliquots of 50mL until use in an incubator at 37°C, the tubing passes through a heater set to control the temperature and the observation chamber of the microscope is maintained at 37°C (measurement continuously monitored).


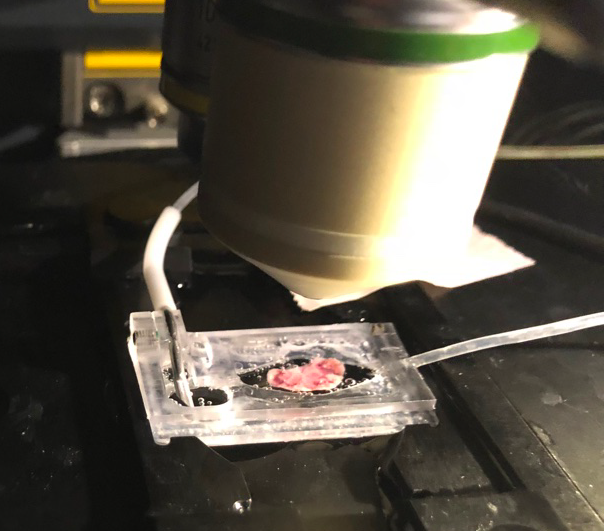


Figure 2 : Device attached to the microscope stage including the glued lung section and the perfusion-aspiration system under the objective.
