# Supplementary material for: Infection with Influenzavirus A in a murine model induces epithelial bronchial lesions and distinct waves of innate immune-cell recruitment
Source: Front Immunol. 2023 Aug 15;14:1241323. doi: 10.3389/fimmu.2023.1241323 (PMC10464834; doi:10.3389/fimmu.2023.1241323)
Supplement: Supplementary file 3 [file DataSheet_3.docx]

| 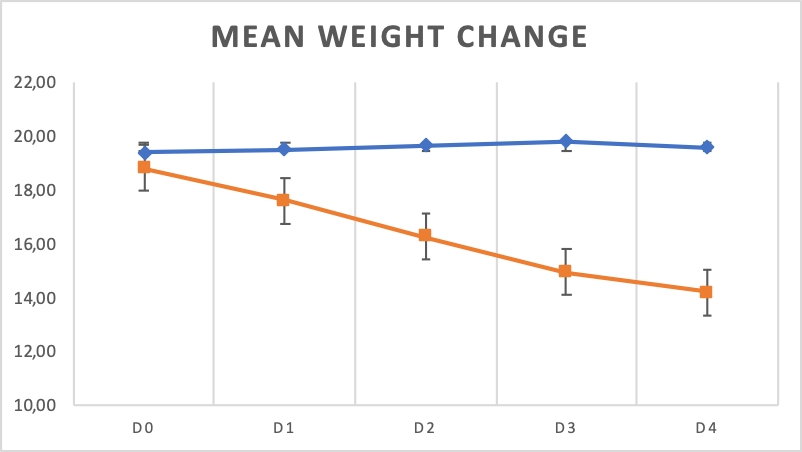 |
| --- |
| Changes in the average (mean ±standard deviation) bodyweight (grams) of mice (n=30) experimentally infected with influenza A (PR8) (orange square) at day 0 compared to six mock-infected animals (no infection) for each time point over a 4-days study (blue diamond). |

Following infection with IAV (PR8), the infected mice group (n=38) lost 25 % of their bodyweight whereas the weight of the control group (n=8) remained stable over the same 4-days period (p<0,05).
